# Assessment of Telomerase Reverse Transcriptase Single Nucleotide Polymorphism in Sleep Bruxism

**DOI:** 10.3390/jcm11030525

**Published:** 2022-01-20

**Authors:** Piotr Macek, Mieszko Wieckiewicz, Rafal Poreba, Pawel Gac, Katarzyna Bogunia-Kubik, Marta Dratwa, Anna Wojakowska, Grzegorz Mazur, Helena Martynowicz

**Affiliations:** 1 Department of Internal Medicine, Occupational Diseases, Hypertension and Clinical Oncology, Wroclaw Medical University, 50-556 Wroclaw, Poland; macekpiotr@op.pl (P.M.); sogood@poczta.onet.pl (R.P.); ania.wojakowska@wp.pl (A.W.); grzegorz.mazur@umw.edu.pl (G.M.); helenamar@poczta.onet.pl (H.M.); 2 Department of Experimental Dentistry, Wroclaw Medical University, 50-425 Wroclaw, Poland; 3 Department of Population Health, Division of Environmental Health and Occupational Medicine, Wroclaw Medical University, 50-345 Wroclaw, Poland; pawelgac@interia.pl; 4 Laboratory of Clinical Immunogenetics and Pharmacogenetics, Hirszfeld Institute of Immunology and Experimental Therapy, Polish Academy of Sciences, 53-114 Wroclaw, Poland; katarzyna.bogunia-kubik@hirszfeld.pl (K.B.-K.); marta.dratwa@hirszfeld.pl (M.D.)

**Keywords:** sleep bruxism, telomerase reverse transcriptase, single nucleotide polymorphism

## Abstract

Introduction: Sleep bruxism (SB) is a widespread masticatory muscle activity during sleep and affects approximately 13.2% of the general population. Telomerase reverse transcriptase (TERT) plays a role in preventing the shortening of the telomere. This prospective, observational study aimed to investigate the relationship between single nucleotide polymorphism (SNP) of TERT and the severity of SB and to identify the independent risk factors for SB. Methods: A total of 112 patients were diagnosed by performing one-night polysomnography based on the guidelines of the American Academy of Sleep Medicine. TERT SNP was detected by real-time quantitative polymerase chain reaction (qPCR). Results: Statistical analysis showed the lack of relationship between the rs2853669 polymorphism of TERT and severity of SB (*p* > 0.05). However, the study showed that patients with allele T in the 2736100 polymorphism of TERT had a lower score on the phasic bruxism episode index (BEI). Based on the receiver operating characteristic (ROC) curve, the value of phasic BEI was 0.8 for the differential prediction for the presence of allele T in the locus. The sensitivity and specificity were 0.328 and 0.893, respectively. The regression analysis showed that lack of TERT rs2736100 T allele, male gender, and arterial hypertension are the risk factors for the higher value of phasic BEI. Conclusion: The SNP of the TERT gene affects phasic SB intensity. The absence of TERT rs2736100 T allele, male sex, and arterial hypertension are independent risk factors for phasic SB.

## 1. Introduction

The widely accepted international consensus by Lobbezoo et al. defines sleep bruxism (SB) as a “masticatory muscle activity during sleep that is characterized as rhythmic (phasic) or nonrhythmic (tonic) and is not a movement disorder or a sleep disorder in otherwise healthy individuals” [1]. The prevalence of SB is estimated to be 13.2% in the general population [2]. The pathogenesis of SB is complex and not fully understood. Age, chronic stress, sensitivity to emotional stress [3] or anxiety disorders, insomnia, caffeine consumption, gastroesophageal reflux disease [4], smoking, alcohol intake, and drug use are considered as relevant risk factors for SB [5]. Polymorphism of dopamine and serotonin receptor genes, dysfunction of the autonomic nervous system [6,7], hypertension [8], and airway obstruction [9] are also associated with the pathogenesis of SB [10].

A recent study showed that SB is associated with systemic inflammation [11], which has been shown to affect telomere length [12]. Telomerase reverse transcriptase (TERT) prevents the shortening of telomeres by synthesizing telomeric repeats onto the end of the 3′ G-rich strand. Telomerase consists of a catalytic subunit TERT, a template ribonucleic acid (RNA), telomerase RNA component (TERC), and several other proteins [13]. Telomeres protect the ends of chromosomes from degradation and end-to-end fusion, thereby providing genomic stability [14,15]. Telomere shortening is associated with loss of proliferative activity and cell aging [16]. When telomeres are damaged or become critically short, DNA damage signaling is triggered, leading to various diseases of aging [17], such as loss of immune function, leukemias, myelodysplastic syndrome, squamous cell skin and gastrointestinal cancers, pulmonary fibrosis, gastrointestinal disorders, liver cirrhosis, and neuropsychiatric conditions [18,19]. Accelerated aging may be usually accompanied by diabetes, hypertension, atherosclerosis, myocardial infarction, graying of hair, and skin pigmentation [20]. Sleep deprivation, delayed circadian rhythm [21], insomnia, and sleep breathing disorder have also been associated with shorter telomeres and alteration in TERT activity [22]. However, there are no data on the relationship between masticatory muscle activity during sleep and TERT gene polymorphism. TERT rs2736100 and TERC rs12696304 are two well-studied single nucleotide polymorphisms (SNPs) that affecting telomere length and/or telomerase expression [23,24,25,26]. Therefore, the present study explored genotypes for the SNPs rs2736100 and rs2853669 of the TERT gene in patients with SB. The present study aimed to investigate the relationship between SNPs of the TERT gene and the severity of SB and to identify the independent risk factors for SB. We hypothesize that the polymorphism of the TERT gene may affect the intensity of SB and that it will be a new and unknown link in the pathogenesis of sleep bruxism.

## 2. Materials and Methods

### 2.1. Participants

The prospective, observational study was conducted in the Department and Clinic of Internal, Occupational Diseases, Hypertension, and Clinical Oncology at the Wroclaw Medical University in Poland. This study was carried out as a part of a project titled “Assessment of telomerase gene polymorphism in patients with obstructive sleep apnea” conducted in Wroclaw Medical University. One-night video polysomnography (PSG) was performed in the Sleep Laboratory of the Wroclaw Medical University by using Nox-A1 (Nox Medical, Reykjavik, Iceland). A total of 112 patients, 55 men and 57 women, were enrolled in the study. The number of patients participating in the study was estimated on the basis of the sample size calculator assuming the following calculation conditions: population size 3 million, fraction size 0.5, confidence level 95%, maximum error 10%. The number of patients recruited for the study exceeded the minimum value of 96 people. Diabetes, hypertension and ischemic heart disease were diagnosed in 9 (8.03%), 35 (31.25%), and 5 (4.46%) participants, respectively. The group characteristic and mean polysomnographic indices are presented in Table 1.

The study was approved by the Ethics Committee of the Wroclaw Medical University (ID KB—525/2020) and was conducted following the Declaration of Helsinki. All the participants provided informed consent to participate in the study. The participants were qualified to enter our study based on the following inclusion criteria: willingness to participate, age > 18 years, and clinically suspected SB and/or obstructive sleep apnea (OSA).The exclusion criteria were as follows: secondary bruxism associated with neurological conditions; intake of drugs that affect neuromuscular functioning, presence of severe mental disorders that cause inability to undergo PSG; active malignancy, respiratory and/or cardiac insufficiency, and active inflammation. The enrolled patients were diagnosed with possible or probable SB according to international concensus by Lobbezoo [1]. There were no patients with definite sleep bruxism diagnosed prior to psg examination.

### 2.2. PSG

The American Academy of Sleep Medicine (AASM) standards were used to diagnose SB. Electromyographic (EMG) recordings were obtained from the masseter muscle region bilaterally. The EMG phenotypes of SB were qualified as phasic, tonic, or mixed. Audio and video recordings were obtained and assessed. Bruxism episode index (BEI) indicates the total number of SB (phasic, tonic, and mixed) episodes per hour. According to the AASM standards, the peak of the EMG amplitude during a bruxism episode must be at least twice the amplitude of the background EMG. If the interruption between episodes was less than 3 s, such interruptions were considered to be part of the same episode [27]. The phasic episode was scored if it lasted at least 2 s with three or more bursts. The episode was qualified as a tonic episode if it lasted for more than 2 s with sustained bursts; a mixed episode was considered if the episode could not be scored as tonic or phasic. SB is diagnosed if the value of BEI is at least 2. We assessed SB’s severity as follows: BEI = 2–4, mild/moderate SB; BEI > 4, severe SB [27]. All patients underwent full-night PSG. Electroencephalogram (EEG) of the patients was recorded using the AASM recommended EEG montages during the PSG. The respiratory effort was assessed based on respiratory inductance plethysmography (RIP) belts circulating the thorax and abdomen. A single modified electrocardiogram Lead II was used to assess ECG. A nasal pressure transducer was used to measure respiratory episodes. Polysomnograms were scored in 30-s epochs. An automatic analysis was conducted, followed by a manual analysis by a certified polysomnographist. Epochs were classified based on the standard criteria for sleep by the AASM 2013 Task Force [28]. The PSG outcomes included the following: sleep latency (SL); wake after sleep onset (WASO); rapid eye movement (REM) latency; total sleep time; sleep efficiency; and the ratio of N1 (sleep stage 1), N2 (sleep stage 2), N3 (sleep stage 3), and REM (REM sleep stage).

Blood samples were collected from the participants by venipuncture at 7.00 AM on the day after PSG.

### 2.3. DNA Extraction

Genomic DNA was isolated from 200 μL of peripheral blood collected in EDTA tubes using the NucleoSpin Blood (MACHEREY-NAGEL GmbH & Co. KG, Dueren, Germany) according to the manufacturer’s instructions. DNA concentration and purity were quantified on a DeNovix spectrophotometer (DeNovix Inc., Wilmington, DE, USA). Isolated DNA was used for TERT genotyping in patients with SB.

### 2.4. Genotyping of TERT Gene Polymorphisms 

The selection of the investigated SNPs in the TERT gene was based on the results from the SNP Function Prediction tool of the National Institute of Environmental Health Sciences (NCBI Database) website and other auxiliary databases (https://snpinfo.niehs.nih.gov/snpinfo/snpfunc.html (last accessed on 1 June 2021); https://www.ncbi.nlm.nih.gov/snp/ (last accessed on 1 June 2021); https://www.ensembl.org/index.html (last accessed on 5 July 2021)). The following criteria were used: minor allele frequency in Caucasians above 10% and change in RNA and/or amino acid chain, potential splicing site, and/or miRNA binding site. 

Based on the above criteria, 2 SNPs were selected for the study: TERT rs2736100 (G > T) located in intron 2, and TERT rs2853669 (T > C), located at −245 bp (Ets2 binding site) in the promoter region. Both TERT rs2736100 and rs2853669 SNPs were determined using LightSNiP typing assays (TIB MOLBIOL, Berlin, Germany). Both assays are based on qPCR. Amplifications were performed on a LightCycler 480 II Real-Time PCR system (Roche Diagnostics International AG) according to the manufacturer’s protocol. The PCR conditions were as follows: 95 °C for 10 min followed by 45 cycles of 95 °C for 10 s, 60 °C for 10 s, and 72 °C for 15 s. PCR was followed by one cycle of 95 °C for 30 s, 40 °C for 2 min, and gradual melting from 75 °C to 40 °C.

### 2.5. Statistical Analysis

The statistical package “Dell Statistica 13.1” (Dell Inc., Round Rock, TX, USA) was used to perform statistical analysis. The arithmetic means and SDs of the estimated parameters were calculated for the quantitative variables. The distribution of variables was examined using Lilliefors test and W-Shapiro–Wilk test. For the independent quantitative variables with normal and non-normal distribution, we used Student’s *t*-test and Mann–Whitney U test, respectively. The results for qualitative variables were expressed as percentages. McNemar’s or Cochran’s test was used for statistical analysis of dependent qualitative variables. To determine the relationship between the analyzed variables, a regression analysis was performed. Parameters of the model obtained in the multivariate regression analysis were estimated using the least squares method. Moreover, the test accuracy was assessed based on ROC (receiver operating characteristic) analysis. The results were considered to be statistically significant at 2-sided *p* < 0.05.

## 3. Results

SB (BEI > 2) was diagnosed in 62 (55.35%) participants. Mild/moderate (2 ≤ BEI < 4) and severe SB (BEI ≥ 4) were confirmed in 22 (19.64%) and 40 (35.71%) subjects, respectively. 

Mild (5 ≤ AHI < 15), moderate (15 ≤ AHI < 30), and severe OSA (AHI ≥ 30) were diagnosed in 24 (21.42%), 20 (17.85%), and 24 (21.42%) subjects, respectively. OSA was excluded (AHI < 5) in 44 (39.28%) participants.

The prevalence of TERT SNPs is presented in Table 2.

In our study, we did not find statistically significant differences between patients with SB (BEI > 2) and without SB (BEI < 2) for SNPs in the TERT gene. 

Our study indicated significant differences between phasic BEI among patients with rs2736100 TERT polymorphism carrying allele T. Phasic BEI (2.06 vs. 3.97, *p* < 0.05) was higher in patients without allele T. We did not observe similar differences in participants with rs2853669 polymorphism of the TERT gene.

The parameters of BEI and phasic, tonic, and mixed episodes in participants carrying allele T and allele G of rs2736100 TERT polymorphism are shown in Table 3.

**Figure 1:**
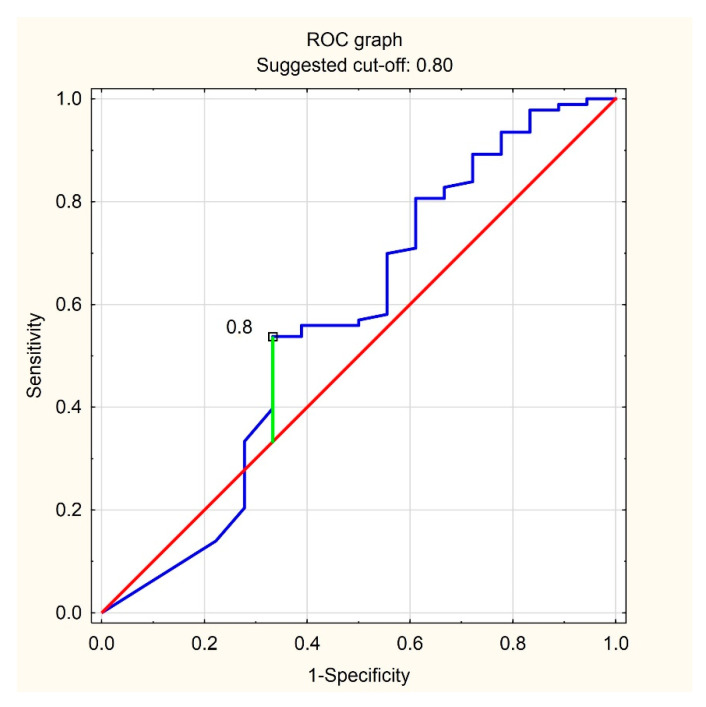
ROC graph.

Because of the significant difference between phasic BEI (2.06 vs. 3.97) in patients for the presence of T allele in the locus of the TERT gene, the receiver operating characteristic (ROC) curve was generated. Based on the ROC curve, the value of PhE/h = 0.8 was determined as the differentiating value for the prediction of the presence of T allele in the studied locus (Figure 1). 

In the subgroup of patients with T allele in the locus of the TERT gene, more participants showed phasic BEI below 0.8 than those without T allele (Table 4).

In the subgroup, the value of BEI below 0.8 indicated the presence of T allele in the locus of the TERT gene with sensitivity and specificity of 0.328 and 0.893, respectively, which gives a prediction accuracy of 0.659. 

A regression analysis was performed to determine the independent factors associated with phasic BEI value. In the univariate regression analysis, the relationships between potential independent variables, i.e., anthropometric parameters, comorbidities, polysomnographic parameters and SNP polymorphisms of telomerase reverse transcriptase, and the dependent variable, i.e., phasic BEI, were determined. Then, by means of backward multivariate regression analysis, the final estimation of the relationship between the variables significant in the univariate analyzes and the phasic BEI was made. The following statistically significant estimation model was obtained: phasic BEI = 5.35 − 2.53 alleles T + 1.63 male gender + 1.23 AH ± 3.37. On the basis of this model, it can be stated that the absence of T allele in the locus, male gender, and arterial hypertension were independent predictors for the increased phasic BEI value in the studied patients.

## 4. Discussion

The most important result of the present study is the association of phasic bruxism episodes and TERT gene polymorphism. The TERT gene is located on chromosome 5p15.33 [29] and encodes the catalytic subunit of telomerase [30,31,32,33]. We investigated two SNPs: rs2853669 in the TERT promoter and rs2736100 located within intron 2 of TERT [34]. The selection of the investigated SNPs in the TERT gene was based on the results from the SNP Function Prediction tool of the National Institute of Environmental Health Sciences. We considered the following criteria: minor allele frequency in Caucasians above 10% and change in RNA and/or amino acid chain, potential splicing site, and/or miRNA binding site.

TERT rs2736100 C allele has been previously shown to be associated with shorter telomere [35], lung cancer [36,37], and sporadic idiopathic pulmonary fibrosis [38,39,40]. Multiple oncogenic roles of TERT in cancer development and progression have been established. However, recently, there is evidence on the protective role of TERT in neurons. TERT may activate autophagy for toxic neuronal proteins and ameliorate the effects of amyloid-β, pathological tau, and α-synuclein involved in neurodegenerative diseases such as Alzheimer’s disease and Parkinson’s disease [41,42,43,44]. TERT is expressed in several regions of the brain, such as the olfactory bulb, cortex [45], hippocampus [46], and subventricular zone and in Purkinje neurons of the cerebellum [47]. Interestingly, caffeine, a known risk factor for SB, may promote TERT expression [48]. It is worth noting that overexpression of TERT in experimental models induces repetitive behaviors and other autism spectrum disorder (ASD)-like behavioral symptoms as well as synaptic deficits [49]. A recent study in an animal model showed that TERT overexpression distorts the balance between excitation and inhibition, impairs hippocampal synaptic plasticity, and reduces the expression of learning-related molecules in the hippocampus [50]. Therefore, we propose that the activity of TERT may affect the neurotransmission and, thus, induce sleep rhythmic masseter movement activity. However, this hypothesis requires further investigation.

We also demonstrated that male gender and arterial hypertension are independent risk factors for phasic SB. Male gender and hypertension were previously reported as risk factors for increased BEI [8]. Thus, the present study is in agreement with these results.

Our study did not indicate the association between tonic or mixed bruxism and SNP of the TERT gene. Firstly, there is very modest information regarding EMG subtypes of SB. It is worth noting that phasic bruxism is the most common SB subtype [51,52]. Tonic bruxism episodes are the rarest; however, in healthy subjects, tonic bruxism is more common [53]. Increased severity of phasic bruxism was observed in hypertensives, while the tonic activity was similar in hypertensives and normotensives [54]. Michalek–Zrabkowska et al. recently showed that snoring intensity correlates with phasic bruxism, but not with tonic bruxism [52]. Thus, phasic bruxism activity seems to be not only the most common, but is also associated with systemic diseases. We demonstrated an association between phasic bruxism and SNP of the TERT gene. Therefore, our result suggests that the pathogenesis of phasic episodes may differ from that of the tonic ones. However, further polysomnographic studies are required to confirm this assumption.

The last, but not least, issue to discuss is whether TERT polymorphism affects cell aging in patients with SB. TERT rs2736100 affects telomere length and/or telomerase expression [54]; therefore, this SNP of the TERT gene may influence the process of aging. Moreover, the phenomenon of telomere shortening in short-term sleepers or those with low sleep quality has been reported. Based on data from a Lebanese population, Zgheib et al. assessed whether the relative telomere length could act as a potential biomarker for age-related diseases. A total of 497 subjects participated in their study. Regression analysis showed that people with sleep difficulty have shorter telomeres than those with normal sleep [55]. Tempaku et al. indicated in their study the association between short telomere length with long sleep duration and insomnia [56]. The increased number of arousals and altered sleep architecture in subjects with SB have been reported previously [57]. Thus, we can hypothesize that the aging process may be altered in sleep bruxers. However, further studies on telomere length and telomerase activity in SB are required to verify this hypothesis.

Summarizing, we have confirmed the hypothesis that polymorphism of TERT may affect SB intensity. However, we have observed the effect on the most common phasic bruxism, but not mixed or tonic.

The main limitation of our study is one-night PSG. It is known that the addition of a second night provides more valuable information; however, because of the Polish national health service regulations, we could not conduct two-night PSG.

Moreover, the studied group was heterogeneous; many of the participants suffered from sleep apnea. It has been repeatedly demonstrated that OSA is a risk factor for SB and that these sleep conditions co-occur frequently. However, through regression analysis, we have shown that TERT polymorphism is a risk factor for phasic bruxism independent of the AHI value.

## 5. Conclusions

(1) The SNP of the TERT gene affects phasic SB intensity.
(2) The absence of TERT rs2736100 T allele, male sex, and arterial hypertension are independent risk factors for phasic SB.
(3) Further studies are needed to assess the role of SNP of the TERT gene and TERT activity in SB pathogenesis.

## Figures and Tables

**Table 1:** Group characteristics and mean values of polysomnographic indices.

Parameter	Mean	Median	Minimum	Maximum	SD
BMI	28.54	28.00	20.00	54.00	5.44
Age	46.27	45.50	20.00	78.00	15.49
AHI (n/h)	16.48	8.75	0.00	100.10	19.48
SL (min)	22.06	15.40	0.00	112.60	21.04
WASO (min)	48.93	33.80	1.00	195.50	41.35
SE (%)	82.90	85.40	52.40	98.30	10.31
N1 (% of TST)	4.94	3.30	0.10	36.10	5.15
N2 (% of TST)	46.68	48.20	15.20	75.80	10.09
N3 (% of TST)	25.98	25.60	2.60	54.90	10.47
REM (% of TST)	22.42	23.10	4.10	38.40	7.65
Arousals (n/h)	5.80	3.30	0.00	51.00	8.01
Obstructive apneas (n/h)	5.77	0.40	0.00	76.90	13.31
Mixed apneas (n/h)	0.31	0.00	0.00	25.20	2.40
Central apneas (n/h)	0.56	0.10	0.00	15.10	1.79
Cheyne-Stokes breathing (% of TST)	0.56	0.00	0.00	15.80	1.93
ODI (n/h)	16.31	9.30	0.00	83.40	18.77
Mean SpO_2_ (%)	93.39	93.75	83.30	97.30	2.42
Minimal SpO_2_ (%)	84.22	85.00	54.00	95.00	7.94
Average Desat Drop (% of TST)	4.31	3.65	2.10	19.80	2.23
BEI (n/h)	4.18	2.35	0.00	24.70	4.58
Phasic BEI (n/h)	2.35	0.75	0.00	19.30	3.39
Tonic BEI (n/h)	1.05	0.70	0.00	6.40	1.17
Mixed BEI (n/h)	0.64	0.45	0.00	4.00	0.70

AHI, apnea–hypopnea index; BMI, body mass index; ODI, oxygen desaturation index; TST, total sleep time (min); SL, sleep latency; REML, REM latency; WASO, wake after sleep onset; SE, sleep efficiency; N1, sleep stage 1; N2, sleep stage 2; N3, sleep stage 3; REM, rapid eye movement sleep stage; mean SpO_2_, mean oxygen saturation; minimal SpO_2_, minimal oxygen saturation; BEI, bruxism episode index; SD, standard deviation.

**Table 2:** Prevalence of TERT SNPs in the entire study group.

TERT rs2736100			TERT rs2853669		
Genotype	*n*	%	Genotype	*n*	%
TG	54	48.65	TT	63	56.76
TT	39	35.14	TC	41	39.94
GG	18	16.21	CC	7	6.3
Allele			Allele		
T	93	83.78	C	48	43.24
G	72	64.86	T	104	93.69

**Table 3:** Polysomnographic parameters of patients carrying allele T and allele G of rs2736100 TERT polymorphism.

SB Parameter	T Allele −	T Allele +	*p*	G Allele −	G Allele +	*p*
BEI (n/h)	6.57 ± 7.2	4.13 ± 6.44	0.15	4.54 ± 8.99	4.52 ± 4.90	0.99
Phasic BEI (n/h)	3.97 ± 5.53	2.06 ± 2.75	**0.02**	2.25 ± 3.33	2.44 ± 3.46	0.78
Tonic BEI (n/h)	1.30 ± 1.64	1.00 ± 1.08	0.33	0.79 ± 0.68	1.19 ± 1.35	**0.08**
Mixed BEI (n/h)	0.87 ± 0.98	0.59 ± 0.63	0.11	0.57 ± 0.54	0.67 ± 0.77	0.48

BEI, bruxism episode index; values are shown as mean ± SD; statistically significant differences (*p* < 0.05) marked as bold.

**Table 4:** Frequency of phasic BEI values depends on the presence of T allele in the studied locus.

	T Allele +	T Allele −
Phasic BEI ≤ 0.8	50 (67%)	6 (33%)
Phasic BEI ≥ 0.8	43 (46%)	12 (67%)

## Data Availability

The data that support the findings of this study are available on request from the corresponding author and are not publicly available due to privacy or ethical restrictions.
